# The involvement of caspases in the process of nuclear removal during lens fiber cell differentiation

**DOI:** 10.1038/s41420-023-01680-y

**Published:** 2023-10-21

**Authors:** Rifah Gheyas, A. Sue Menko

**Affiliations:** 1 Department of Pathology and Genomic Medicine, Sidney Kimmel Medical College, Thomas Jefferson University, Philadelphia, PA US; 2 Department of Ophthalmology, Sidney Kimmel Medical College, Thomas Jefferson University, Philadelphia, PA US

**Keywords:** Proteolysis, Proteolysis, Differentiation

## Abstract

The terminal differentiation of lens fiber cells involves elimination of their organelles, which must occur while still maintaining their functionality throughout a lifetime. Removal of non-nuclear organelles is accomplished through induction of autophagy following the spatiotemporal suppression of the PI3K/Akt signaling axis. However, blocking this pathway is not alone sufficient to induce removal of fiber cell nuclei. While the final steps in fiber cell nuclear elimination are highlighted by the appearance of TUNEL-positive nuclei, which are associated with activation of the lens-specific DNaseIIβ, there are many steps in the process that precede the appearance of double stranded DNA breaks. We showed that this carefully regulated process, including the early changes in nuclear morphology resulting in nuclear condensation, cleavage of lamin B, and labeling by pH2AX, is reminiscent of the apoptotic process associated with caspase activation. Multiple caspases are known to be expressed and activated during lens cell differentiation. In this study, we investigated the link between two caspase downstream targets associated with apoptosis, ICAD, whose cleavage by caspase-3 leads to activation of CAD, a DNase that can create both single- and double-stranded DNA cleavages, and lamin B, a primary component of the nuclear lamina. We discovered that the specific inhibition of caspase-3 activation prevents both lamin B and DNA cleavage. Inhibiting caspase-3 did not prevent nuclear condensation or removal of the nuclear membrane. In contrast, a pan-caspase inhibitor effectively suppressed condensation of fiber cell nuclei during differentiation. These studies provide evidence that caspases play an important role in the process of removing fiber cell nuclei during lens differentiation.

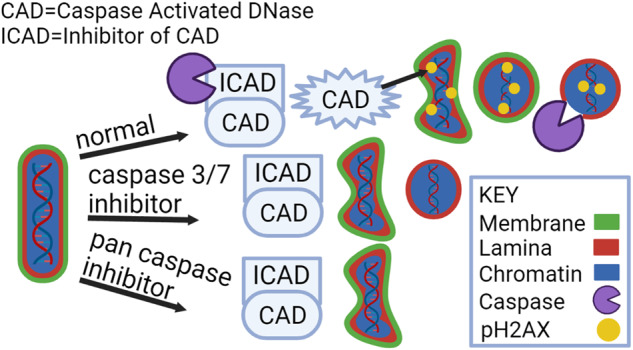

## Introduction

Key to lens function is the differentiation-state-specific elimination of organelles, including nuclei, from its fiber cells. Our previous studies show that the spatiotemporal induction of autophagy through suppression of the PI3K/Akt signaling axis leads to the removal of non-nuclear organelles, but is alone insufficient for nuclear elimination [1–3]. The challenge of removing the nuclei from lens fiber cells while retaining their function throughout a lifetime requires a highly regulated process that remains to be determined. Prior to their removal fiber cell nuclei transition from a highly elongated to a condensed morphology [4, 5], with their ultimate elimination dependent on DNaseIIβ [6]. A component of lysosomes, DNaseIIβ first associates with the perinuclear region [7], and enters the nucleus to cleave chromatin following removal of the nuclear envelope [6–8]. Entry of DNaseIIβ to fiber cell nuclei requires disassembly of the nuclear lamina following CDK1-dependent phosphorylation of lamin A [9], consistent with its function at the latest stages of nuclear elimination. TUNEL-labeling of condensed lens nuclei is observed just prior to their elimination and is linked to DNaseIIβ function, with chromatin retained in DNaseIIβ null mice [6]. It remains to be investigated whether other DNases also function to execute nuclear elimination.

The removal of lens nuclei shares many properties with apoptosis including chromatin condensation, DNA cleavage, and disassembly of the nuclear envelope. All are linked to caspase function including caspase-3/7, with caspase-3 functioning in activation of Caspase Activated DNase (CAD) to cleave chromatin [10, 11] and caspase-6, which mediates nuclear shrinkage [12]. These caspases are activated through either the intrinsic mitochondrial death pathway or the extrinsic death receptor pathway. Cleavage of nuclear proteins by executioner caspases promotes terminal differentiation of erythroblasts and keratinocytes [13, 14]. Caspase activation is associated with both lens differentiation initiation and organelle loss [15–18]; we have investigated their role in nuclear elimination.

In apoptosis caspase-3 cleaves the Inhibitor of Caspase Activated DNase (ICAD), leading to the release and activation of Caspase Activated DNase (CAD) [19–24], which can create either single- or double-stranded breaks [25]. pH2AX is recruited to both of these cleavage sites [26]; TUNEL only detects double-stranded DNA breaks [27]. The activation of caspase-3 during lens fiber cell differentiation [17, 18, 28, 29] suggests that its role in the induction of CAD may be important during early stages of lens fiber cell nuclear elimination. Additionally, caspase-3 targets intermediate filaments, including the nuclear lamins [30, 31], which form the nuclear lamina, the cytoskeletal meshwork associated with the inner nuclear membrane providing structural support to the nucleus [30–32]. The lamina, together with the nuclear membrane, comprise the nuclear envelope. LAP2, an integral membrane protein of the inner nuclear membrane binds directly to lamin B, linking the nuclear membrane to the nuclear lamina [33]. Both lamin B and LAP2 bind to chromatin and are essential to organizing chromatin in interphase cells [33, 34].

In this study, we investigated whether the role of caspases in cleaving the nuclear lamina and chromatin has an essential function in the elimination of lens fiber cell nuclei during their differentiation. Our results showed that caspase-3 regulates both nuclear condensation and removal. The specific inhibition of caspase-3 impairs both nuclear elimination and fragmentation of the nuclear lamina, while blocking all caspase activity, including caspase-6, in differentiating lens fiber cells also suppresses nuclear condensation.

## Results

### Distinct spatiotemporal pattern of removal of the nuclear membrane and lamina

**Fig. 1:**
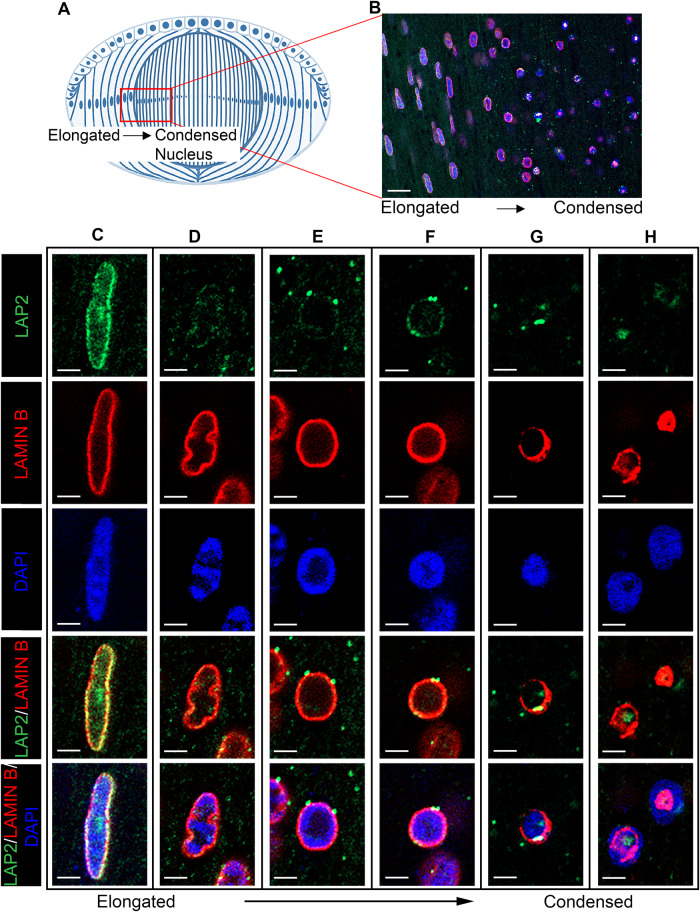
Differentiation-state specific morphological changes in lens fiber cell nuclei. **A** Model depicting the process of nuclear loss in the embryonic chick lens at E14, with the red boxed-in area the region of interest imaged in **B**. Cryosections of E14 lenses were co-immunolabeled for the nuclear transmembrane protein LAP2 (green) and the nuclear lamina protein lamin B (red), co-labeled with DAPI to localize chromatin (blue). **B** Single optical plane from a 40× z-stack acquired by confocal microscopy in the region of the lens where nuclei transition from elongated to condensed. Scale bar, 20 μm. **C**–**H** Zoomed in regions of the different stages of nuclear elimination from the image shown in **B**, taken at single optical planes from the acquired z-stack. The first three rows show single channels of LAP2, lamin B and chromatin. The fourth row is an overlay of LAP2 and lamin B, the fifth row an overlay of LAP2, lamin B, and chromatin. Scale bar, 2 μm.

We investigated the mechanisms by which lens fiber cells are able to remove their nuclei while maintaining the ability to function throughout a lifetime. Our approach began with performing a detailed analysis of the spatiotemporal changes to both the nuclear membrane and the nuclear lamina relative to the timing of chromatin condensation and elimination. This study examined chick embryo lenses at embryonic day 14 (E14), a developmental time at which fiber cells are present at each stage of nuclear removal including the transition from an elongated to a condensed morphology (modeled, Fig. 1A). Lens cryosections were co-immunolabeled for the transmembrane inner nuclear membrane protein LAP2 and the nuclear lamina cytoskeletal protein lamin B, and counter-labeled with the DNA-binding fluorescent stain DAPI. Confocal image analysis was performed, and Z-stacks were collected, with images presented of a single optical plane. An overview is presented at low magnification (Fig. 1B) along with high magnification images of individual nuclei at different stages of the elimination process (Fig. 1C-H).

The fiber cells of the lens cortex have elliptical shaped nuclei to which lamin B and LAP2 co-localized at their outer edges (Fig. 1C). Chromatin was distributed throughout these nuclei and enhanced along the lamina (Fig. 1C). As the fiber cells differentiate, labeling for lamin B highlighted loss of the nuclear elliptical shape, characterized by the wavy appearance of the lamina (Fig. 1D), as reported previously [4]. Chromatin was organized in clusters that sometimes appeared directly linked to the nuclear lamina (Fig. 1D). LAP2, a nuclear membrane marker, was greatly diminished, with fragments that remained co-localizing with lamin B (Fig. 1D). This finding demonstrated that the membrane compartment of the nuclear envelope is lost early in fiber cell differentiation, and much before the lamina. The undulating cytoarchitecture of the nuclei at this stage of fiber cell differentiation is a transition state that precedes nuclear condensation in more differentiated fiber cells. When lens fiber cell nuclei become condensed, their structural support is still provided by a lamin B-rich cytoskeleton to which chromatin is highly marginalized (Fig. 1E). As fiber cell nuclei continued to condense, chromatin became more widely distributed across the lamin B encapsulated nuclei (Fig. 1F). The nuclear lamina became fragmented as fiber cells matured in the central-most regions of the developing lens, with significant regions missing around the condensed chromatin (Fig. 1G). Just prior to nuclear loss, residual fragments of the lamin B cytoskeleton and LAP2 appeared to aggregate, with chromatin extending beyond their boundaries (Fig. 1H). These findings provided evidence that the changes in nuclear morphology prior to nuclear elimination are governed by alterations to the nuclear lamina [30].

### Development-state specific targeting of nuclear envelope components

**Fig. 2:**
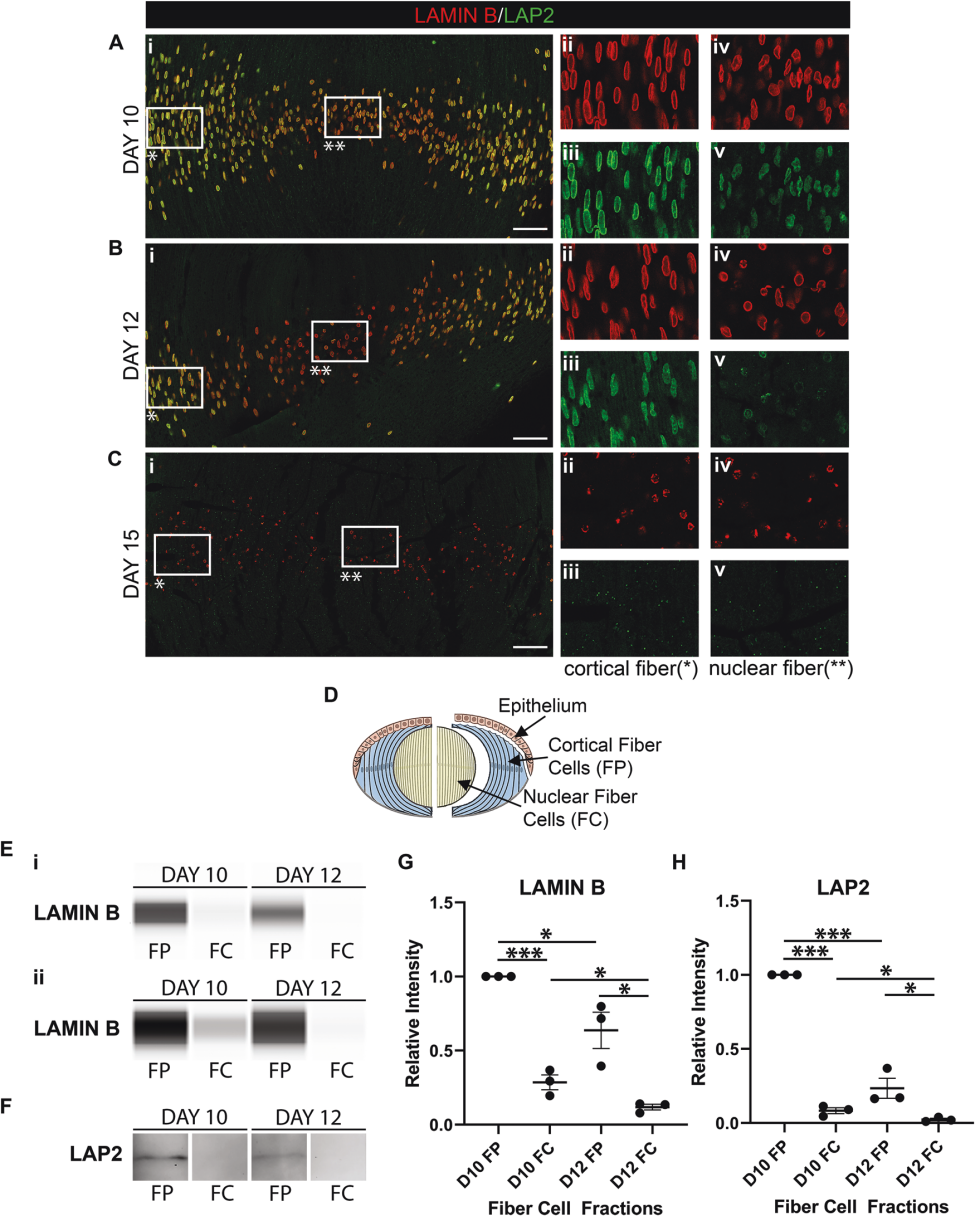
Development-specific patterns of nuclear membrane and nuclear lamina loss that precede nuclear elimination. **A**–**C** Lens cryosections were immunolabeled for lamin B (red) and LAP2 (green) at **A** E10, **B** E12, and **C** E15. (Ai-Ci) 40× confocal tile acquired in the region of lens fiber cell differentiation. (Aii, iii-Cii, iii) Zoomed in view of the boxed in regions of Ai-Ci denoted by a single asterisk, (Aii-Cii) lamin B, (Aiii-Ciii) LAP2. (Aiv, v-Civ, v) Zoomed in view of the boxed in regions of Ai-Ci denoted by a double asterisk, (Aiv-Civ) lamin B, (Av-Cv) LAP2. Data shows loss of the nuclear membrane precedes the loss of the nuclear lamina. Scale bar, 50 μm. Results representative of 3 independent studies. **D** Model of microdissection of lens fiber regions to yield cortical fiber (FP) and central fiber (FC) cell fractions. FP and FC regions of the embryo lens at E10 and E12 were immunoblotted for **E** lamin B, analyzed using the WES and **F** LAP2, using standard Western Blot analysis. Eii represents a higher exposure of the immunoblot shown in Ei. Quantifications, performed on three independent studies, are shown for both **G** lamin B and **H** LAP2. Error bars represent S.E.M, *P ≤ 0.05, **P ≤ 0.01, and ***P ≤ 0.001, *t*-test.

Lenses were examined at E10, E12, and E15, stages prior to nuclear removal, after the process is initiated, and during active elimination, to determine the developmental timing of removal of the nuclear membrane and lamina. Cryosections were co-immunolabeled for LAP2 and lamin B, counterstained with DAPI, and imaged by confocal microscopy. Results are presented at low magnification of LAP2/lamin B (Fig. 2Ai, Bi, Ci) and lamin B/DAPI (Supplemental Fig. 1) overlays acquired across the entire lens fiber cell region, and for LAP2 and lamin B individually at high magnification in both the cortical and central fiber cell zones of differentiation (Fig. 2Aii-v, Bii- v, Cii-v). The nuclei of cortical fiber cells at E10 remained highly elongated and elliptical, with both LAP2 and lamin B strongly co-localized at their periphery (Fig. 2Aii, iii). There are already changes to membranes of central fiber cell nuclei at E10, which have begun to change shape, evidenced by loss of LAP2 (Fig. 2Av). Their nuclei retain their lamin B-rich lamina (Fig. 2Aiv). By E12, LAP2 labeling revealed extensive removal of the membrane from nuclei in the center of the lens (Fig. 2Bv), with this loss of LAP2 now extending into fiber cells of the adjacent lens cortex (Fig. 2Biii). Central fiber cell nuclei at E12 were condensed, some retaining lamin B all along their periphery, others in which the nuclear lamina was fragmented (Fig. 2Biv). In the adjacent cortical fiber cells, the undulating morphology of lamin B along the nuclear periphery revealed the beginnings of the dynamic nuclear remodeling process (Fig. 2Bii). By E15, the time of active nuclear removal, the nuclear membrane was lost from central and cortical fiber cells (Fig. 2Ciii,v). Moreover, chromatin retained by central fiber cells had little to no associated lamina (Fig. 2Civ), and the lamina was fragmented in cortical fiber cell nuclei (Fig. 2Cii). A consistent feature of nuclear elimination throughout lens development is the loss of the nuclear membrane prior to the nuclear lamina.

Immunoblot analysis was performed on microdissected cortical (FP) and central (FC) fiber cell fractions (modeled Fig. 2D) from E10 and E12 lenses for both lamin B (Fig. 2Ei-ii, G) and LAP2 (Fig. 2F, H). Lamin B results are shown at two different exposures, the higher to highlight lamin B in central fiber cell nuclei (Fig. 2Eii). The results confirmed the loss of lamin B in cortical (FP) and central (FC) lens fiber cells between E10 and E12 (Fig. 2Ei-ii, G). They revealed that while lamin B remains a major component of the nuclear lamina surrounding the chromatin of the central lens fiber cells at E10, the changes in nuclear morphology occurring just prior to condensation resulted in a reduction of lamin B protein (Fig. 2Ei, G). Immunoblot for LAP2 showed the early loss of the nuclear membrane from central fiber cells, and the significant loss of LAP2 from the nuclear envelope of cortical fiber cells between E10 and E12 (Fig. 2F, H).

### Nuclear lamina breakdown precedes appearance of double-stranded DNA breaks

**Fig. 3:**
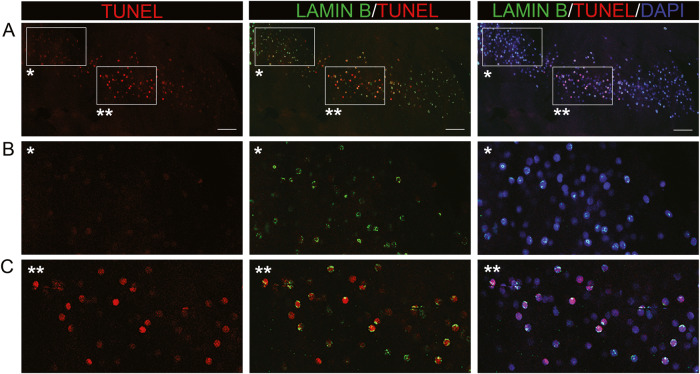
Breakdown of the nuclear lamina precedes appearance of TUNEL-positive nuclei. E15 lens cryosections were labeled by TUNEL assay (red), and co-labeled with antibody to lamin B (green) and the nuclear dye DAPI (blue). **A** 40× confocal tiles were acquired across the region of lens fiber cell differentiation and panels (left to right) show TUNEL labeling alone, TUNEL/lamin B overlay, and TUNEL/lamin B/DAPI overlay. Boxed in regions in A denoted with a single asterisk in each column are shown at higher magnification in **B**, and boxed in regions in A denoted with a double asterisk in each column are shown at higher magnification in **C**. Scale bar, 50 μm. Results representative of 3 independent studies.

The differentiation state-specific appearance of double-stranded DNA breaks in lens fiber cells was examined relative to breakdown of the nuclear lamina at E15. No TUNEL labeling of central fiber cells was detected prior to E15. TUNEL-labeled lens cryosections were co-labeled for both lamin B and nuclei, and low magnification confocal images acquired across the lens (Fig. 3A), with cortical and central fiber cell zones also shown at higher magnification (Fig. 3B, C). No double-stranded DNA breaks were detected in cortical fiber cells even if their nuclei had already become condensed (Fig. 3B), and their lamina fragmented or removed (Fig. 3B). In contrast, the condensed nuclei in the center of the E15 lens were TUNEL positive, often with fragments of the nuclear lamina linked to their periphery (Fig. 3C). These studies showed that breakdown of the nuclear envelope occurs much earlier in fiber cell differentiation than the creation of double stranded DNA breaks.

### DNA cleavage precedes TUNEL labeling of fiber cell nuclei

**Fig. 4:**
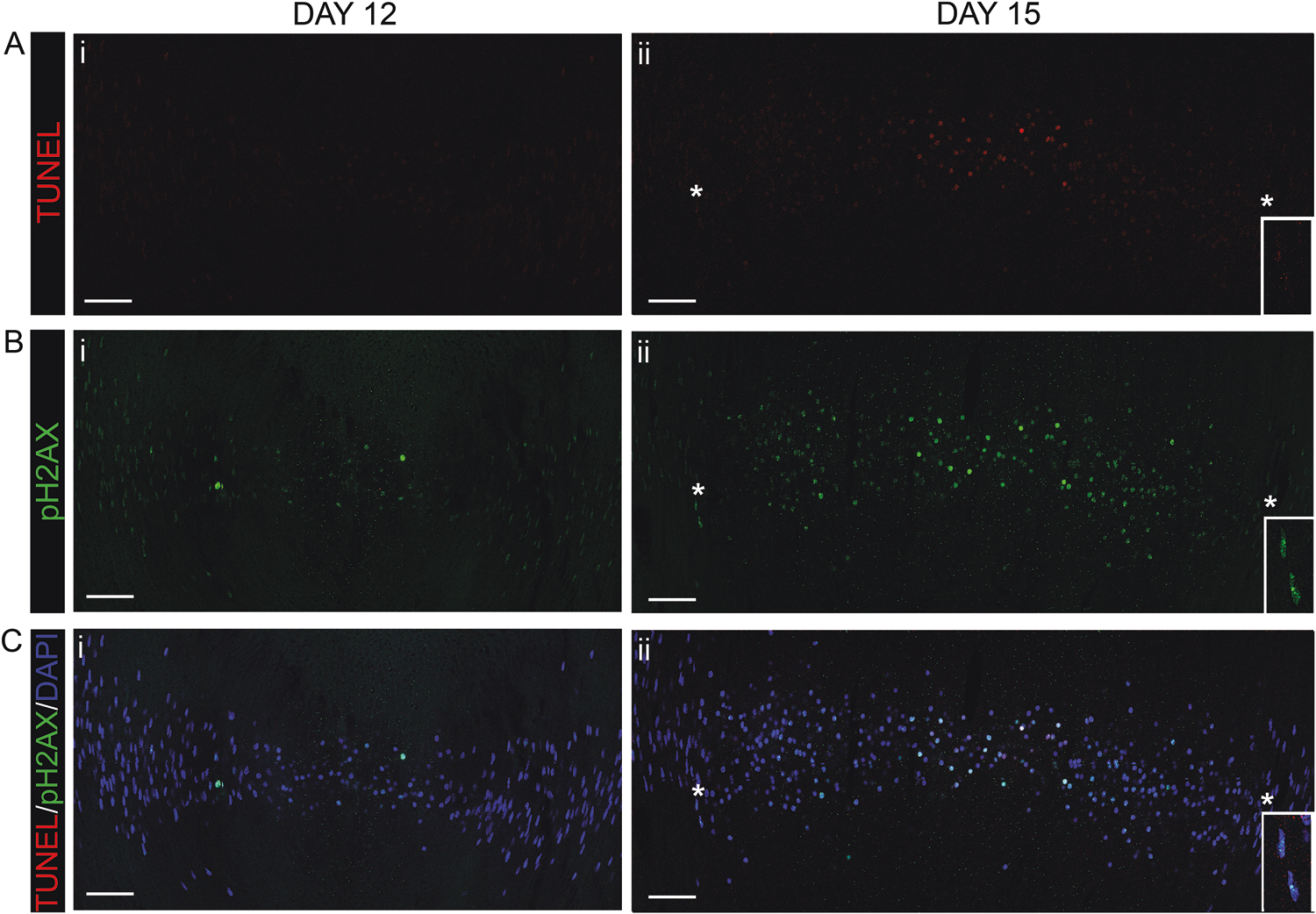
DNA cleavage precedes nuclei becoming TUNEL-positive during the lens fiber cell differentiation process. Ai–Ci E12 and Aii–Cii E15 lens cryosections were labeled by TUNEL assay (red), and co-immunolabeled with antibody to pH2AX (green), and the nuclear dye DAPI (blue). **A**–**C** Images are 40× tiles acquired by confocal microscopy across the lens fiber cell differentiation region, with data shown as **A** TUNEL, **B** pH2AX, and **C** TUNEL/pH2AX/DAPI overlay. The insets in Aii-Cii show a higher magnification of the nuclei denoted by an asterisk, highlighting that pH2AX, but not TUNEL, labels nuclei prior to their condensation. Since TUNEL assay labels double stranded DNA breaks and pH2AX can label both single and double stranded breaks, these results suggest that single-stranded breaks are executed in lens fiber cell chromatin prior to more significant fragmentation of lens DNA that accompanies nuclear elimination. Scale bar, 50 μm. Results are representative of three independent studies.

The histone H2AX becomes phosphorylated (pH2AX) in response to both single- and double- stranded DNA damage [26, 35]. Therefore, to investigate the spatiotemporal cleavage of fiber cell DNA further, TUNEL-stained lens cryosections were immunolabeled with antibody to pH2AX at both E12 and E15 (Fig. 4). Confocal microscopy imaging revealed the presence of pH2AX+ nuclei in central fiber cells as early as E12 (Fig. 4Bi, Ci), a developmental time prior to the appearance of double-stranded DNA cleavages detected by TUNEL assay (Fig. 4Ai, Ci). TUNEL labeling was not detected until E15, and then only in the condensed, center-most fiber cell nuclei (Fig. 4Aii, Cii). In contrast, pH2AX labeled both cortical and central fiber cell nuclei at E15 (Fig. 4Bii, Cii). These results suggest that single-stranded DNA cleavage events executed by one or more DNases other than DNaseIIβ must precede the DNA cleavage carried out by DNaseIIβ for the process of fiber cell nuclear elimination during lens development.

### Caspase activated DNase imported to the nucleus prior to nuclear condensation

Our data supports the conclusion that multiple DNases must be involved in cleaving fiber cell chromatin as these cells prepare to eliminate their nuclei during their final stages of differentiation. To identify likely DNases that could play this role, we queried a RNAseq database, GEO ascension number is GSE53976, that was created from four differentiation- specific regions of the E13 chick embryo lenses we had isolated by microdissection. These regions included undifferentiated epithelial cells (EC), the region of the epithelium where lens cell differentiation is initiated (EQ), differentiating cortical fiber cells (FP), and central fiber cells undergoing the final stages of maturation (FC). We discovered that message for both CAD and its inhibitor ICAD are expressed in all regions of the lens, increasing significantly in lens fiber cells (Supplemental Fig. 2). CAD/ICAD are co-translated as an inactive complex [36], and CAD is activated following ICAD cleavage by caspase-3 [37–39]. The activation of CAD can occur either in the cytoplasm [40] or after import of the ICAD/CAD complex to the nucleus [19, 21].

**Fig. 5:**
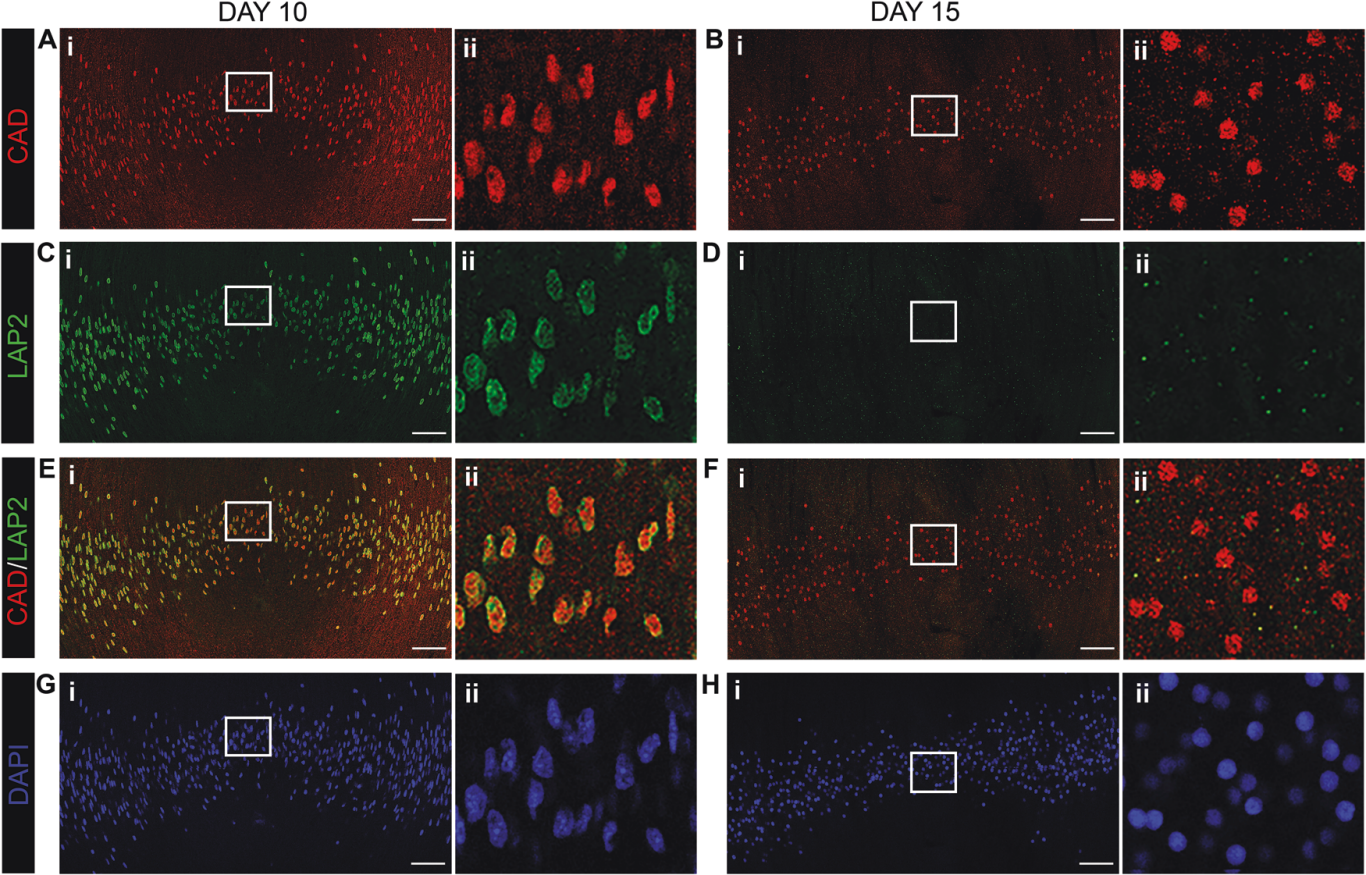
CAD localizes to the nuclei of differentiating lens fiber cells while their nuclear membrane is still intact. **A**, **C**, **E**, **G** E10 and **B**, **D**, **F**, **H** E15 lens cryosections were co-immunolabeled for CAD (red) and LAP2 (green), with chromatin labeled using DAPI (blue). **A**–**H** Images are 40× tiles acquired by confocal microscopy across the lens fiber cell differentiation region, with data shown as **A**, **B** CAD, **C**, **D** LAP2, **E**, **F** CAD/LAP2 overlay, and **G**, **H** DAPI label alone. The boxed-in regions in the center of the lens in Ai-Hi are shown at higher magnification to the right in Aii-Hii, respectively. The results show that at the early developmental time of E10, prior to nuclear condensation, CAD is localized to fiber cell nuclei whose nuclear membrane is intact. Scale bar, 50 μm. Results representative of 3 independent studies.

Lens differentiation- and development-stage specific nuclear localization of CAD was examined relative to degradation of the nuclear membrane by co-immunolabeling for CAD and LAP2 at E10 and E15 (Fig. 5A–H). Confocal image analysis revealed that CAD was imported to fiber cell nuclei by E10, prior to both nuclear condensation (Fig. 5Ai-ii, Ei-ii, Gi-ii) and nuclear membrane fragmentation (Fig. 5Ci-ii, Ei-ii). This is in contrast to DNaseIIβ, which gains access to the chromatin only after removal of the nuclear envelope [6, 41]. At E15, CAD was localized to discrete puncta in the remaining central fiber cell nuclei (Fig. 5Bi-ii, Fi-ii), a labeling pattern observed as early as E12 (Supplemental Fig. 3).

**Fig. 6:**
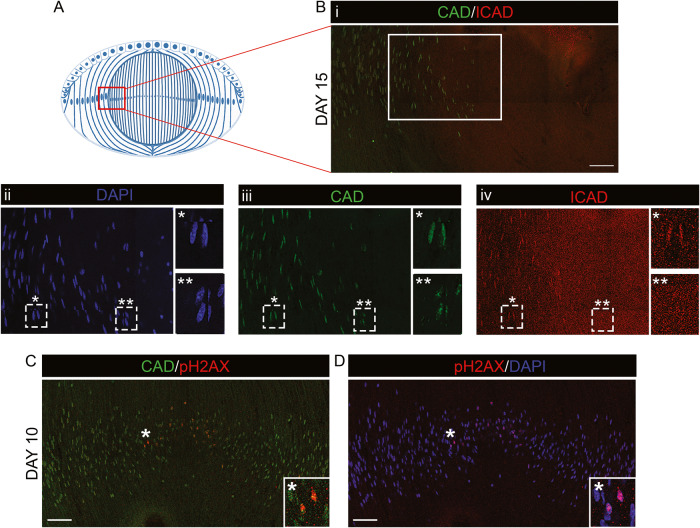
ICAD is lost from fiber cell nuclei just prior to nuclear condensation. **A** Model depicting the process of nuclear loss in the embryonic chick lens at E15, with the red boxed-in area the region of interest imaged as a 40× confocal tile in **B**i. (**B**i-iv) E15 lens cryosections co- immunolabeled for CAD (green) and ICAD (red), with chromatin labeled using DAPI (blue). (**B**i) CAD/ICAD overlay with the boxed in area in Bi shown at higher magnification for each label in (**B**ii) DAPI, (**B**iii) CAD, and (**B**iv) ICAD. (**B**ii-**B**iv) The dashed line boxed-in region where fiber cell nuclei have a highly elongated morphology (single asterisk) and in the region just prior to nuclear condensation (double asterisk) are shown at higher magnification in the panels to the right. The loss of ICAD in the nuclei of fiber cells bordering the central fiber zone where nuclei become condensed suggests CAD is activated prior to nuclear condensation. **C**, **D** E10 lens cryosection co-immunolabeled for CAD (green) and pH2AX (red) and co-stained with DAPI (blue), images acquired as a 40× tile by confocal microscopy across the central region of lens fiber cells; **C** CAD/pH2AX overlay, **D** DAPI/pH2AX overlay. Region denoted by asterisk is shown at higher magnification in the inset. The pH2AX labeling of elongated central fiber cell nuclei at E10 demonstrates that chromatin cleavage occurs at this early stage of fiber cell differentiation, prior to nuclear condensation. Scale bar, 50 μm. Results representative of 3 independent studies.

As ICAD cleavage by caspase-3 releases and activates CAD, lens differentiation state-specific CAD activation was determined by co-labeling lens cryosections for ICAD, CAD, and DAPI at E15, a developmental time when all stages of nuclear removal are present (Fig. 6A, B). For ICAD/CAD co-labeling, the CAD antibody was conjugated to a 488 fluorophore, which interfered with its ability to bind to CAD in the center-most, highly condensed fiber cell nuclei. The results revealed that CAD and ICAD were co-localized in the elongated nuclei of the cortical fiber zone (Fig. 6Biii-iv, single asterisks), and that ICAD but not CAD was lost from fiber cell nuclei as they undergo the morphological restructuring that occurs prior to nuclear condensation and elimination (Fig. 6Biii-iv, double asterisks). Therefore, CAD, but not ICAD, labeled nuclei whose morphologies are intermediate between elongated and condensed, consistent with the activation of CAD in differentiating lens fiber cell nuclei prior to their condensation.

Our studies have shown that DNA cleavage (pH2AX labeling) precedes TUNEL labeling of differentiating fiber cell nuclei (Fig. 4). To examine the relationship between CAD activation and the early cleavage events of lens fiber cell chromatin, E10 lens cryosections were co-immunolabeled for CAD and pH2AX. CAD co-localized with pH2AX in many of the nuclei in the center of the E10 lens (Fig. 6C), showing that spatiotemporal-specific chromatin cleavage events, either single or double stranded, were already occurring in the embryonic lens at early stages of development. These results support the conclusion that in addition to DNaseIIβ, CAD, and possibly other DNases, play active roles in chromatin remodeling in preparation of nuclear elimination to form the lens Organelle Free Zone.

### Caspases play a regulatory role in elimination of fiber cell nuclei

Caspases target two pathways important to the process of nuclear degradation, the activation of CAD and the degradation of proteins in the nuclear lamina. To examine the link between caspases and the processes involved in fiber cell nuclear condensation and elimination, E10 lenses were exposed in organ culture to either the pan caspase inhibitor Z-VAD-FMK (100 μM) or the caspase-3 specific inhibitor Z-DEVD-FMK (200 μM) for 48 h, with DMSO as control. The inhibitors effectively blocked lens caspase activity (Supplemental Fig. 4).

**Fig. 7:**
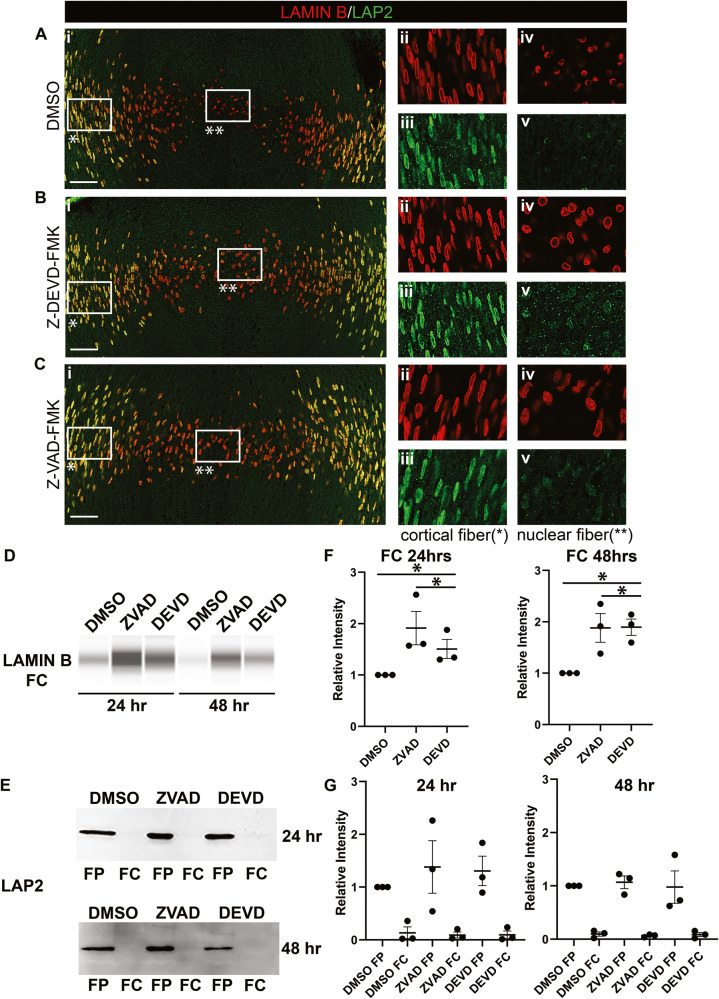
Caspase inhibition prevents disassembly of the nuclear envelope, impairing nuclear condensation. **A**–**C** Cryosections of E10 lenses exposed for 48 h in organ culture to **A** DMSO, vehicle control, **B** the caspase 3 inhibitor Z-DEVD-FMK, or **C** the pan-caspase inhibitor Z-VAD-FMK, co-immunolabeled for lamin B (red) and LAP2 (green). (**A**i-**C**i) Lamin B/LAP2 overlay, images acquired as 40x tiles by confocal microscopy across the region of lens fiber cell differentiation. (Aii, iii-Cii, iii) Zoomed in view of the boxed in regions of Ai-Ci denoted by a single asterisk, (**A**ii-**C**ii) lamin B, (**A**iii-**C**iii) LAP2. (**A**iv, v-**C**iv, v) Zoomed in view of the boxed in regions of Ai-Ci denoted by a double asterisk, (**A**iv-**C**iv) lamin B, (**A**v-**C**v) LAP2. Data shows that caspase inhibition suppresses the loss of the nuclear lamina (lamin B) and nuclear condensation without impacting the disassembly of the nuclear membrane. Scale bar, 50 μm. Results are representative of three independent studies. **D**, **E** Fiber cells from E10 lenses exposed for 24 or 48 h in organ culture to DMSO, Z-VAD-FMK, or Z-DEVD-FMK were microdissected to separate cortical fiber cells (FP) from central fiber cells (FC). **D** The FC fractions were immunoblotted for lamin B using WES technology with the results quantified in **F**. **E** Both FP and FC fiber cell fractions were immunoblotted for LAP2 by Western Blot, the results quantified in **G**. Quantifications were performed on three independent studies. Error bars represent S.E.M., *P ≤ 0.05, **P ≤ 0.01, and ***P ≤ 0.001, *t*-test.

The impact of the caspase inhibitors on differentiation state-specific degradation of the nuclear lamina and membrane and the condensation/elimination of lens fiber cell nuclei was determined by confocal imaging of lens cryosections co-labeled for LAP2, lamin B and DAPI (Fig. 7, Supplemental Fig. 5). The nuclear membrane was removed and the lamina fragmented as nuclei were eliminated in central fiber cells of control lens organ cultures (Fig. 7Ai, iv, v; Supplemental Fig. 5), and retained in their cortical fiber cells (Fig. 7Ai-iii), as occurs in vivo. When caspase-3 activity was blocked, the lamina of central fiber cells remained intact but their membrane was removed (Fig. 7Bi, iv; Supplemental Fig. 5). While their nuclei had become condensed, they failed to be eliminated. Exposure of lenses to the pan-caspase inhibitor, which will also block activation of caspase-6, had an even greater impact on the nuclear elimination process by blocking condensation of central fiber cell nuclei (Fig. 7Ci, Supplemental Fig. 5). The nuclear lamina of these cells was retained, but not their nuclear membrane (Fig. 7Ci, iv, v).

The impact of the caspase inhibitors on lamin B and LAP2 in central fiber cell was determined at 24 h and 48 h in lens organ culture by immunoblot analysis. Both caspase inhibitors effectively suppressed degradation of lamin B, the pan-caspase inhibitor with a greater effect than the capase-3 inhibitor (Fig. 7D, F). There was no significant impact on LAP2 degradation (Fig. 7E, G).

**Fig. 8:**
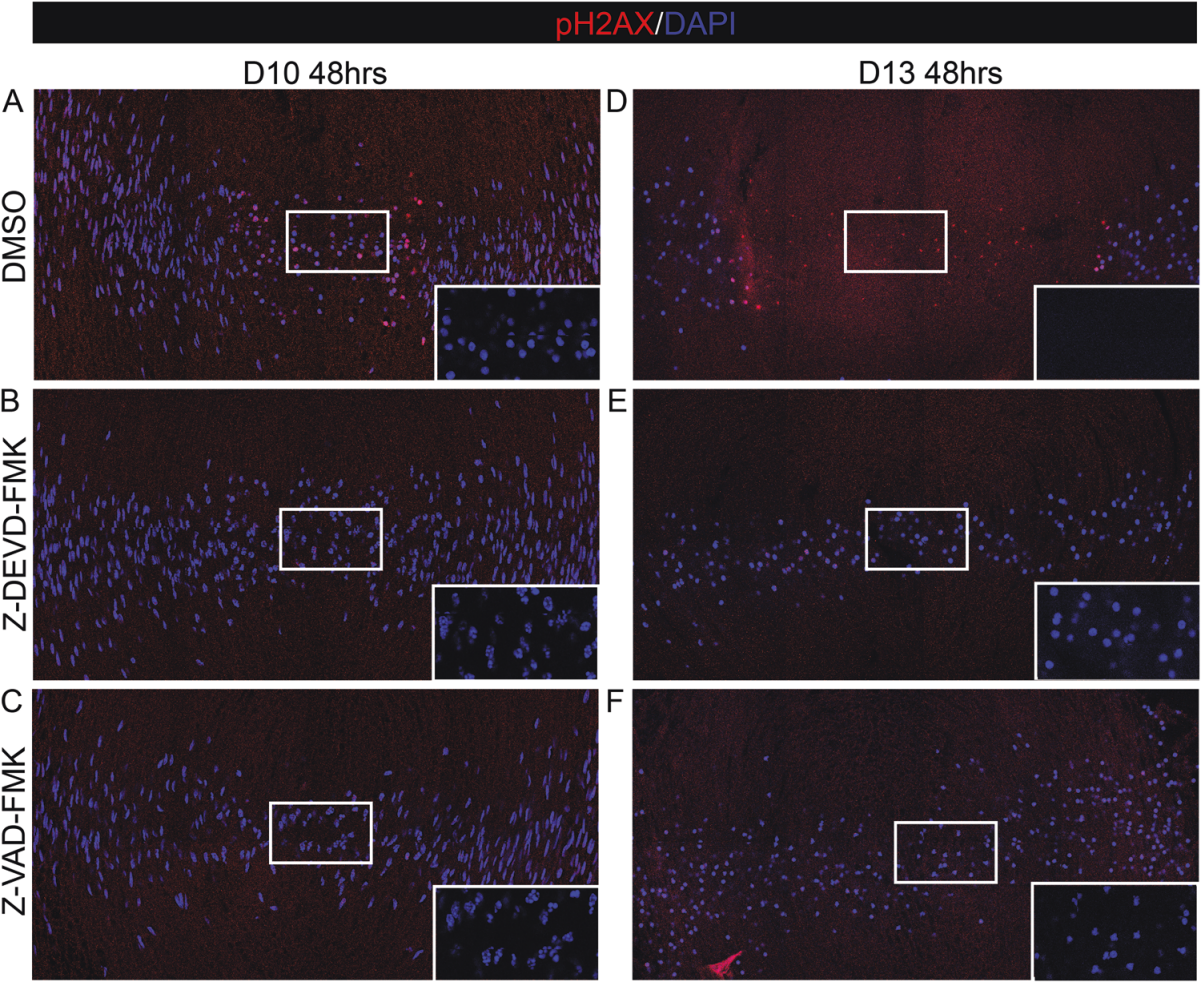
Suppression of caspase activity blocks cleavage of fiber cell DNA, nuclear condensation, and nuclear elimination. Cryosections of **A**–**C** E10 or **D**–**F** E13 lenses exposed for 48 h in organ culture to **A**, **D** DMSO, vehicle control, **B**, **E** the caspase-3 inhibitor Z-DEVD-FMK, or **C**, **F** the pan-caspase inhibitor Z-VAD-FMK, immunolabeled for pH2AX (red) and co-labeled with DAPI (blue). Insets are a higher magnification of DAPI staining alone of the boxed-in region at the center. Blocking caspases at D10 for 48 h suppressed both DNA cleavage and nuclear condensation. Blocking caspases at D13 for 48 h suppressed DNA cleavage and nuclear elimination. Scale bar, 50 μm. Results representative of 3 independent studies.

To determine whether blocking caspase activation interferes with the formation of double stranded DNA cleavages required to execute the final stages of fiber cell nuclear elimination we examined E10 and E13 lenses in organ culture following 48 h of exposure to the caspase inhibitors. The central fiber cell nuclei of control E10 cultures were TUNEL+ (Fig. 8A), and nuclei had been eliminated in E13 controls (Fig. 8D). Exposure to either the caspase-3 specific or pan-caspase inhibitors blocked chromatin cleavage and nuclear elimination in both E10 and E13 lenses (Fig. 8B, C, E, F), but chromatin condensation in central fiber cells was only prevented when lenses were exposed to the inhibitors at E10 (Fig. 8B, C).

## Discussion

Achieving the differentiation-state-specific spatiotemporal removal of lens fiber cell nuclei while maintaining the functionality of these cells throughout a lifetime is a unique challenge, especially considering the many different nuclear components that must be targeted and degraded for nuclear condensation and elimination. Similarities between lens nuclear removal and apoptosis, including caspase-3’s role in lamin B and ICAD cleavage, prompted early studies of caspases as potential regulators of the nuclear removal process, with multiple labs finding evidence that caspases-3, -6, and -7 are expressed/activated in developing and/or adult lenses [17, 18, 28, 42].

Yet, the mouse knockouts of these caspases, either alone or in pairs, failed to prevent complete nuclear loss [17]. Using a different approach, we now show that caspases have multiple essential roles in the elimination of lens nuclei during development.

Lamin A and lamin B are the primary components of the nuclear lamina, providing nuclei with structural support, dictating their shape, and serving as sites for heterochromatin binding [43]. In apoptosis, lamin B is specifically cleaved by caspase-3 [44], while caspase-6 targets lamin A [45], with both contributing to nuclear condensation. The disassembly of lamin A in lens fiber cells following its phosphorylation by Cdk1 has also been linked to creating accessibility of DNaseIIβ to the nucleus for its function in cleaving fiber cell chromatin during the final stages of nuclear elimination [46]. We show that specifically blocking caspase-3 during the formation of the OFZ is sufficient to suppress lamin B degradation, but not nuclear condensation. Exposure to a pan-caspase inhibitor that targets caspase-3 and caspase-6 effectively blocks condensation of lens nuclei. While these distinctions could alone explain our results with different caspase inhibitors, it does not exclude the possibility that caspase targeting of other nuclear molecules also contributes to the impairment of nuclear elimination.

Much of the literature on lens OFZ formation has focused on the role of DNaseIIβ in fiber cell denucleation [6–8, 41]. This DNase, specifically expressed by the lens, is responsible for the double-stranded cleavage of lens fiber cell DNA at the final stages of nuclear elimination. While chromatin was retained in DNaseIIβ null mice, the early steps in the mechanism of nuclear removal, including nuclear condensation and nuclear envelope degradation, occurred normally [6, 7]. This is consistent with our observation in the chick embryo lens model that TUNEL+ nuclei were detected only after nuclei had condensed and the nuclear envelope was lost. We found that pH2AX, which localizes to sites of single- and double-stranded DNA cleavages, became associated with lens nuclei early in fiber cell differentiation, providing strong evidence that other DNases are active in fiber cell nuclei prior to DNaseIIβ. Our studies suggest that ICAD, cleaved by caspase-3 prior to both the removal of the lens fiber cell nuclear envelope and nuclear condensation, activates CAD to create DNA cleavages to which pH2AX is recruited. A similar correlation between caspase-3 activation, ICAD cleavage, and CAD activation for a non-apoptotic role in cell differentiation was first demonstrated in studies of myoblast differentiation. Our findings with the developing lens provide evidence that chromatin alterations are essential early steps in the fiber cell nuclear elimination process.

There are a number of potential candidate upstream inducers of caspase activation for their role in lens fiber cell nuclear elimination, many leading to mitochondrial depolarization and the release cytochrome c to induce the canonical mitochondrial death pathway. We have shown that most lens mitochondria are depolarized during the early stages of lens cell differentiation, with a subset retaining polarity until later in the differentiation process [16]. Since we have found that suppression of all PI3K signaling in the developing lens induces premature removal of lens nuclei [3], it is likely that the upstream inducer of caspase activation for the purpose of lens nuclear elimination involves a pathway that blocks a specific a PI3K downstream effector. It will also be important to determine how the pathway(s) that induces caspase activity to eliminate lens nuclei are regulated in order to prevent their action from leading to fiber cell death. Insight is provided by our previous studies of caspase function during the early stages of lens differentiation, where we showed that caspase activity is maintained at low levels due to an IGF- 1/Nfκb survival-signaling pathway [47]. We predict that the activation of caspases during lens fiber cell differentiation will involve complex regulatory mechanisms to eliminate lens nuclei without leading to fiber cell death.

## Methods

### Animals

Thomas Jefferson University’s Institutional Animal Care and Use Committee (IACUC) approved the animal studies that were performed. The investigations comply with all relevant guidelines. The animal studies also comply with the Association for Research in Vision and Ophthalmology Statement for the Use of Animals in Ophthalmic and Vision Research. Embryonated white leghorn chicken eggs, days 10-15, were obtained from Poultry Futures (Lititz, PA).

### Lens microdissection

Embryonic chicken lenses were removed and microdissected to isolate the epithelial (E), cortical fiber (FP), and nuclear fiber (FC) cell regions at E10, 12, and 15, as described previously [48]. Typically, 10 lenses were used for microdissections performed at each stage of development examined.

### Preparation of ex vivo whole lens organ cultures

Ex vivo lens organ cultures were prepared as described previously [1]. Lenses were placed in 96 well plates, one lens per well, in Complete Medium (Medium 199 [Life Technologies, 11150-059] with 10% fetal bovine serum, 1% penicillin, and 1% streptomycin) at 37 °C. Only lenses that were transparent at 1 h were included in the studies; any lenses that had opacities were excluded. Lenses were exposed for 48 h in organ culture to caspase inhibitors or their vehicle DMSO as control. The inhibitors used in this study included the pan-caspase inhibitor Z-VAD- FMK (100 μM) and the caspase 3 inhibitor Z-DEVD-FMK (200 μM). For immunoblotting the studies used 10 lenses/condition, for immunostaining the studies used three lenses per condition. Each study was performed as three independent experiments. The sample sizes and number of biological replicates were predetermined based on previous immunoblot and immunofluorescence studies on lens organ cultures performed in the laboratory [1, 3, 47]. Inhibitor concentration was chosen based on their effective inhibition of caspase activity in both the more peripheral cortical lens fiber cells and the central lens fiber cells. Note that the inhibitors must cross the thick matrix capsule that surrounds the lens so the effective concentration of inhibitors within lens tissue will be lower than that added to the media.

### Antibodies

Antibodies to lamin B (13435) and pH2AX (2577) were from Cell Signaling Technology. Antibody to CAD (PA5-115114) was from Thermo Fisher Scientific. Antibody to LAP2 (611000) and ICAD (550736) were from BD Biosciences. Secondary antibodies (115-545-003, 111-025- 003, 111-545-003) were from Jackson ImmunoResearch Laboratories. Nuclei were labeled with the DAPI reagent (422801) from Biolegend.

### Fluorescent tagging of the CAD antibody

Before tagging the antibody with a fluorophore, 50 K MWCO Pierce protein concentrators (Thermo Scientific, 88504) were used to dilute the glycerol in the sample. Antibody was then centrifuged at 15000 rpm until about 1% glycerol was remaining. The sample was then diluted back to the original volume with PBS. An Alexa Fluor 488 labeling kit (Thermo Scientific, A20181) was used to conjugate the fluorophore to the antibody.

### Immunoblotting

WES—Simple Western system by ProteinSimple: WES immunoblots were performed as previously described [3]. Briefly, lenses were microdissected into differentiation-state specific regions and extracted in an SDS/urea buffer (2 M urea, 34 mM SDS, and 50 mM Tris-HCl at pH 8 with protease and phosphatase inhibitors). The BCA assay (Thermo) was used to determine the protein concentrations of the extracts. Samples were prepared for WES immunoblot by mixing 3 μgs of protein with a 5× Fluorescent Master Mix and Sample Buffer (ProteinSimple) and then denatured at 100 °C for 5 min. Primary antibodies are diluted in Antibody Diluent II (ProteinSimple) and the secondary antibodies are used undiluted. The biotinylated ladder (5 μl), protein lysates (3 μl), primary antibodies (10 μl), secondary antibodies (10 μl), a luminol- peroxidase mix (15 μl), and wash buffer (500 μl) are loaded into designated wells of a WES plate (ProteinSimple). A set of capillaries (ProteinSimple) and the loaded WES plate are placed into the WES apparatus (ProteinSimple). Data was analyzed using Compass for Simple Western software (ProteinSimple).

### Standard Western blot

Western blots were performed as described previously [1]. Briefly, microdissected lens fractions were extracted in SDS/urea buffer, protein concentration was determined, and 150 μg of the sample loaded into the wells of SDS-PAGE 4–12% precast tris/glycine gels (Invitrogen). Proteins were electrophoretically transferred onto Immobilon-P membranes (Millipore) and blocked in 5% milk for 1 h at room temperature. They were probed with primary antibodies at 4 °C overnight and then incubated with secondary antibodies conjugated to horseradish peroxidase (Bio-Rad) for 2 h at room temperature. Protein was detected using ECL plus reagent (Thermo Fisher Scientific) and imaged using the FluorChem E & M Imager (ProteinSimple). Full, uncropped blots are uploaded as “Supplemental Material”.

### Immunofluorescence

Lenses were removed and fixed for 2 h at 4 °C in 4% paraformaldehyde. They were cryoprotected (30% sucrose) before being frozen in Polyfreeze Tissue Freezing Media Red (Polyscience #25115). Serial cryosections 30 μm thick were cut using a Microm HM 550 Cryostat and central sections were used for the immunolabeling studies. Antigen retrieval was performed before labeling, as described previously [49]. Briefly, the sections were permeabilized with 0.05% Triton X-100 in 1× Tris-buffered saline (TBS) for 10 min and then boiled in sodium citrate buffer for 20 min. After cooling down for 10 min, samples were incubated in block buffer (10% goat serum in 1× TBS with 0.05% Triton X-100) for 1 h at room temperature. Sections were incubated with primary antibodies in buffer containing 1% BSA for either 3 h at 37 °C or overnight at 4 °C, followed by incubation with secondary antibody for 2 h at 37 °C (Jackson ImmunoResearch Laboratories). Nuclei were labeled using the DAPI reagent (Biolegend).

### TUNEL assay

TUNEL assay kit (Sigma Aldrich, 12156792910) was used to label double stranded DNA breaks. Sections were co-labeled with DAPI and with antibody to lamin B or pH2AX.

### Confocal Image Analysis

Confocal Image Analysis Labeled sections were imaged using a Zeiss LSM800 confocal microscope with four laser lines: 405, 488, 561, and 640, on a Zeiss Axio Imager Z2 microscope with a motorized XY scanning stage. All images were acquired using the Zeiss Plan-Apochomat 40×/1.3 Oil objective and analyzed with Zen software. 40× tile z-stacks were acquired with an optical plane thickness of 0.5 μm. Presented images are of a single optical plane.

### Caspase activity assay

Flash frozen microdissected lens fractions were extracted in HEPES buffer (20 mM HEPES, 150 mM NaCl, 1% Triton X-100, 0.1% SDS, 1 mM EDTA, pH 7.5). The BCA protein assay was used to determine the protein concentration of the samples. A Caspase-Glo 3/7 Assay Kit (Promega, G8090) was used to determine the amount of caspase 3/7 activity, performed in a 96 well plate. The plate was read on a FlexStation (Molecular Devices) and analyzed using Softmax software.

### Statistical analysis

Statistical analysis was performed for quantification of the immunoblot results. For this analysis, we determined whether a treatment had an effect between two different groups by using the two-sided, unpaired *t* test on three independent experiments. Error bars represent SEM, which provide variance between samples examined in the immunoblot studies. Variances differ between groups. Center values are presented as mean. Differences were considered significant when *P ≤ 0.05, **P ≤ 0.01, ***P ≤ 0.001.

### Supplementary information

Supplemental Figure Legends

Supplemental Figure 1

Supplemental Figure 2

Supplemental Figure 3

Supplemental Figure 4

Supplemental Figure 5

Original Data File

## Data Availability

Original western blots are uploaded as Supplementary Material.
