# Supplementary material for: The involvement of caspases in the process of nuclear removal during lens fiber cell differentiation
Source: Cell Death Discov. 2023 Oct 21;9:386. doi: 10.1038/s41420-023-01680-y (PMC10590423; doi:10.1038/s41420-023-01680-y)
Supplement: Supplementary file 1 — Supplemental Figure Legends [file 41420_2023_1680_MOESM1_ESM.docx]

**SUPPLEMENTAL**

**Supplemental Figure 1)** Development-state specific nuclear condensation precedes loss of the nuclear lamina. Cryosections of (A) E10, (B) E12, and (C) E15 lenses were immunolabeled for lamin B (red) and co-labeled with DAPI (blue). (A-C) Lamin B/DAPI overlay images were acquired as 40x tiles by confocal microscopy across the region of lens fiber cell differentiation. At E12 when nuclei are condensed, lamin B remains intact. By E15, lamin B has been disassembled but fragments remain associated with chromatin prior to nuclear removal. Scale bar, 50μm. Results representative of 3 independent studies.

**Supplemental Figure 2)** Expression of both CAD and ICAD increases in lens fiber cells. Expression levels of CAD and ICAD were obtained from RNAseq data of E13 chick lenses that were microdissected to yield four distinct regions of differentiation including: EC - undifferentiated lens anterior epithelium, EQ - equatorial epithelium, the site of lens differentiation initiation, FP - nascent, differentiating fiber cells located in the lens cortex, and FC - maturing fiber cells located in the center of the lens ([GSE53976](https://www.ncbi.nlm.nih.gov/geo/query/acc.cgi?acc=GSE53976)) showed that CAD and ICAD are expressed at much higher levels in lens fiber regions than in the lens epithelium.

**Supplemental Figure 3)** Nuclear localization of CAD to differentiating fiber cell nuclei. Cryosections of E12 chick embryo lenses were immunolabeled for CAD (red) and co-labeled for DAPI (blue). 40x confocal tiles were acquired by confocal microscopy across the region of lens fiber cell differentiation. Boxed in regions are shown at higher magnification in the panels on the right. Scale bar, 50μm. Results representative of 3 independent studies.

**Supplemental Figure 4)** Caspase activity assay shows efficacy of caspase inhibitors in the different regions of lens cell differentiation. E10 lenses treated for 24 hours with either the vehicle control DMSO, the caspase 3 inhibitor Z-DEVD-FMK, or the pan-caspase inhibitor Z-VAD-FMK and microdissected to separate epithelial (E), cortical fiber (FP), and central fiber (FC) fractions and assayed for caspase activity. (A) Caspase activity in Z-VAD-FMK-treated lenses relative to controls; (B) Caspase activity in Z-DEVD-FMK-treated lenses relative to controls. The data shows that caspase activity is suppressed in all regions lenses exposed to the caspase inhibitors. Results representative of 3 independent studies.

**Supplemental Figure 5)** Caspase inhibition suppresses degradation of the nuclear lamina and slows nuclear condensation. E10 lens cryosections treated for 48 hrs with (A) DMSO, (B) Z-DEVD-FMK, or (C) Z-VAD-FMK were immunolabeled for lamin B (red) and co-labeled with DAPI (blue). (A-C) Lamin B/DAPI overlay images were acquired as 40x tiles by confocal microscopy across the region of lens fiber cell differentiation. Both caspase inhibitors suppressed lamin B disassembly. Scale bar, 50μm. Results representative of 3 independent studies.
