# Supplementary figures and images for: The involvement of caspases in the process of nuclear removal during lens fiber cell differentiation
Source: Cell Death Discov. 2023 Oct 21;9:386. doi: 10.1038/s41420-023-01680-y (PMC10590423; doi:10.1038/s41420-023-01680-y)

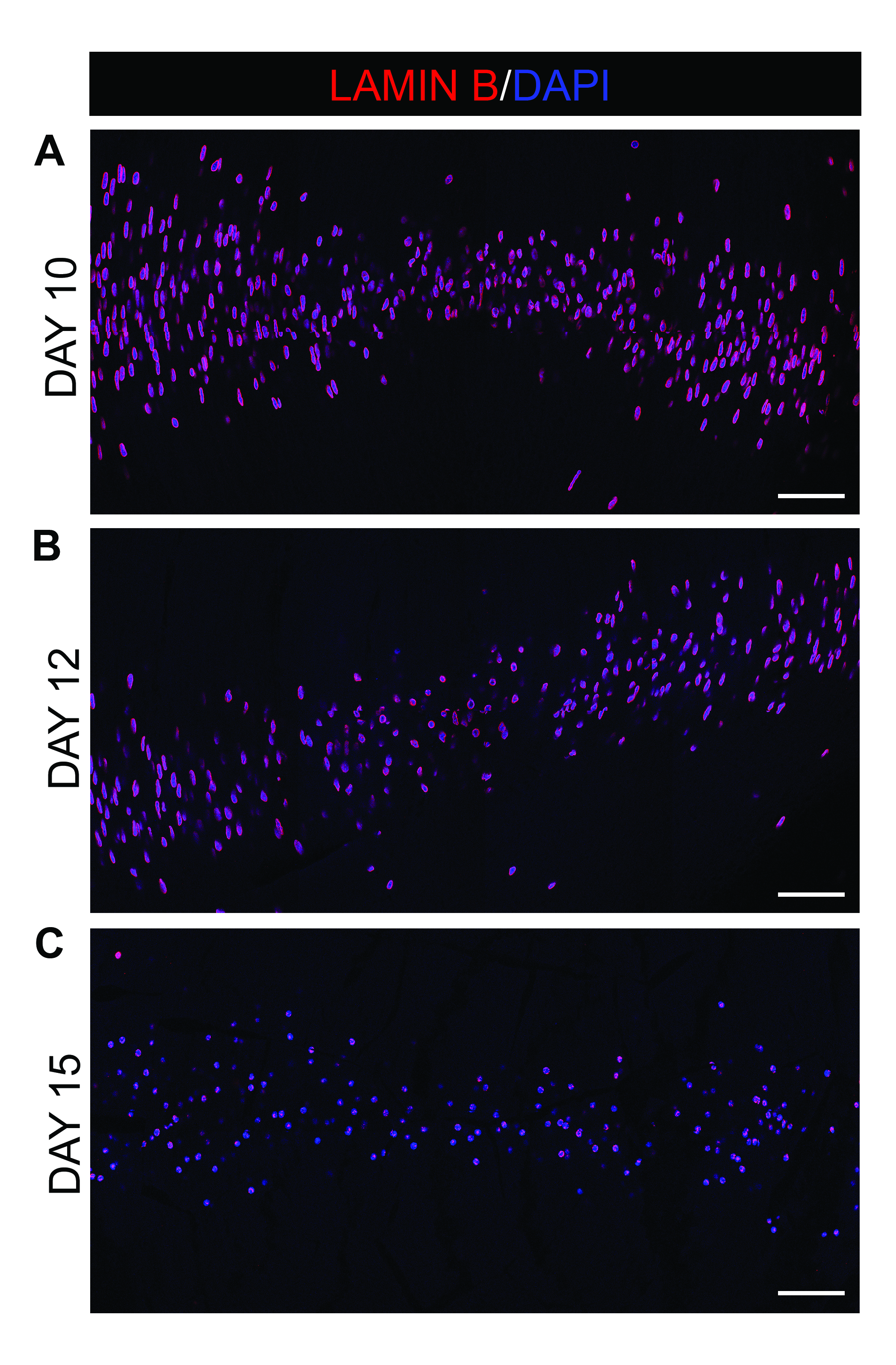

Supplement: Supplementary file 2 — Supplemental Figure 1 [file 41420_2023_1680_MOESM2_ESM.tif]

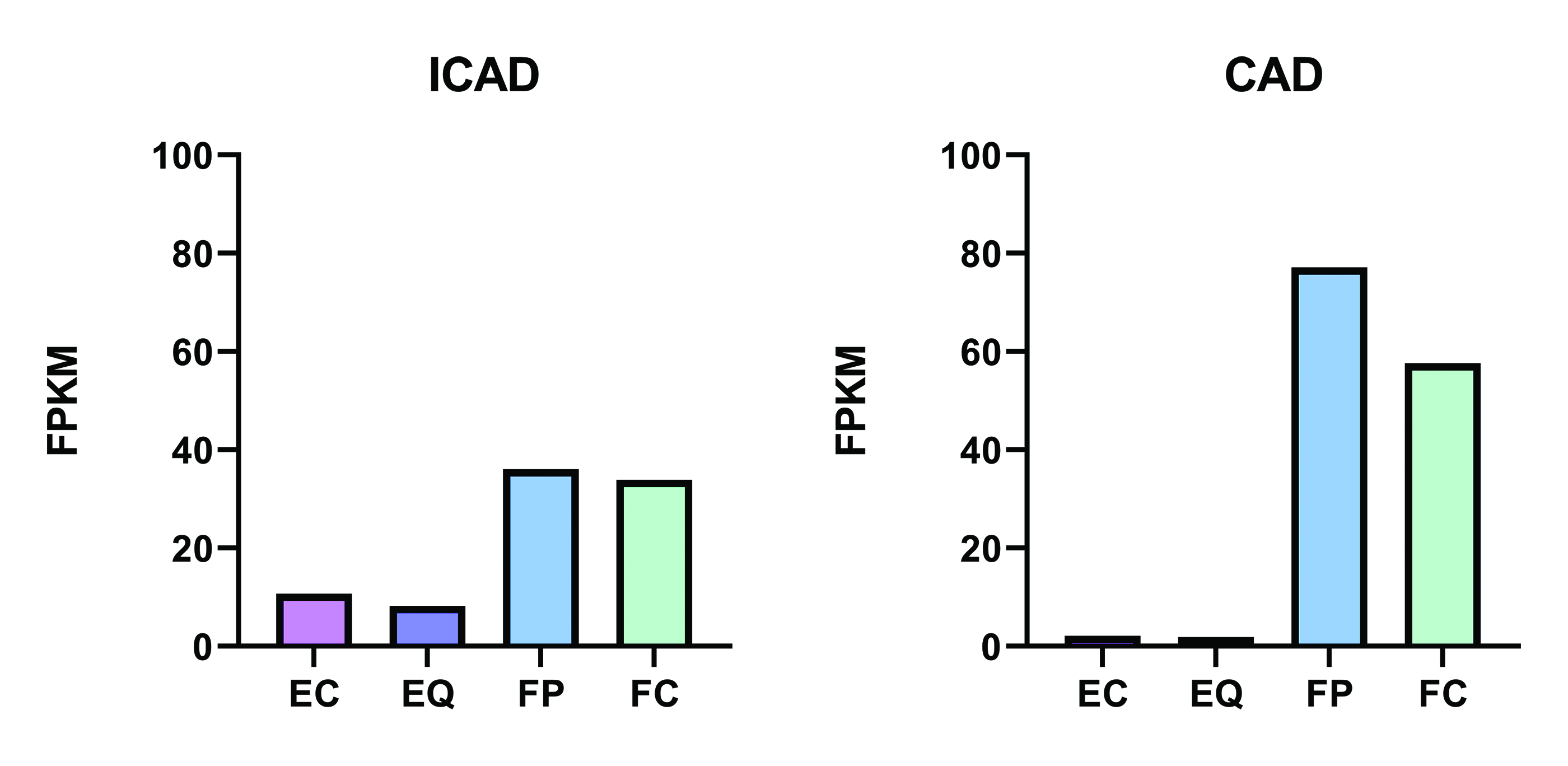

Supplement: Supplementary file 3 — Supplemental Figure 2 [file 41420_2023_1680_MOESM3_ESM.tif]

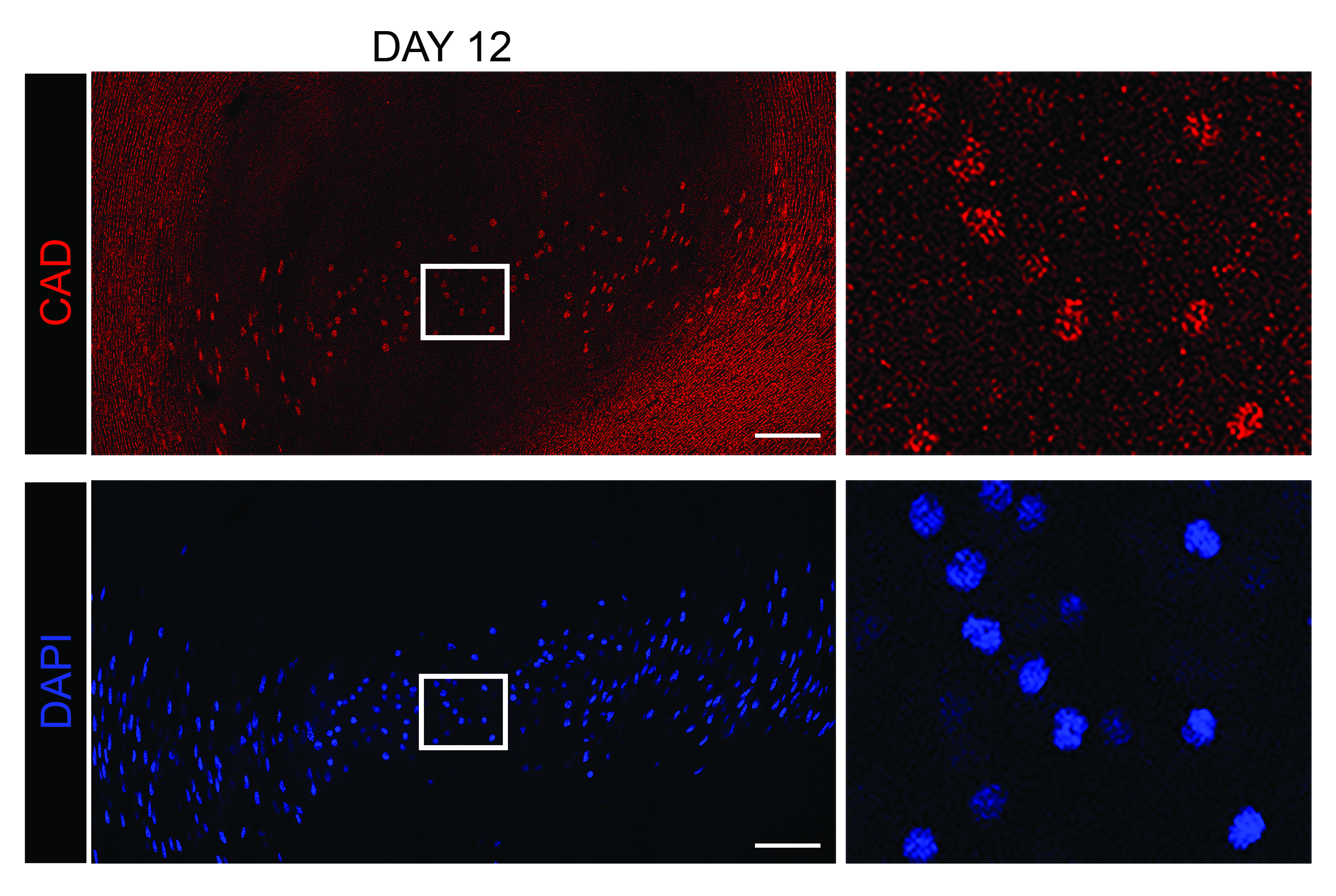

Supplement: Supplementary file 4 — Supplemental Figure 3 [file 41420_2023_1680_MOESM4_ESM.tif]

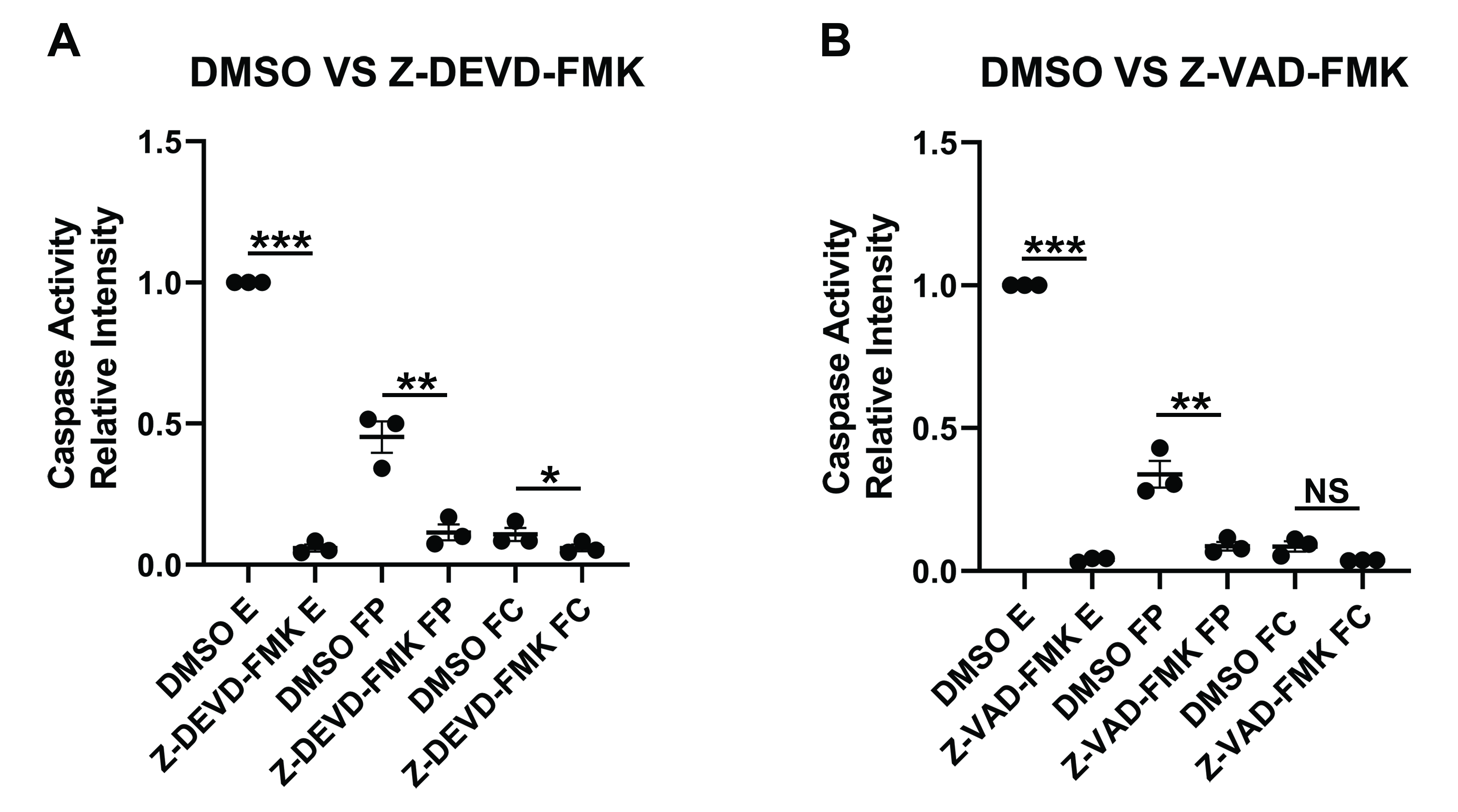

Supplement: Supplementary file 5 — Supplemental Figure 4 [file 41420_2023_1680_MOESM5_ESM.tif]

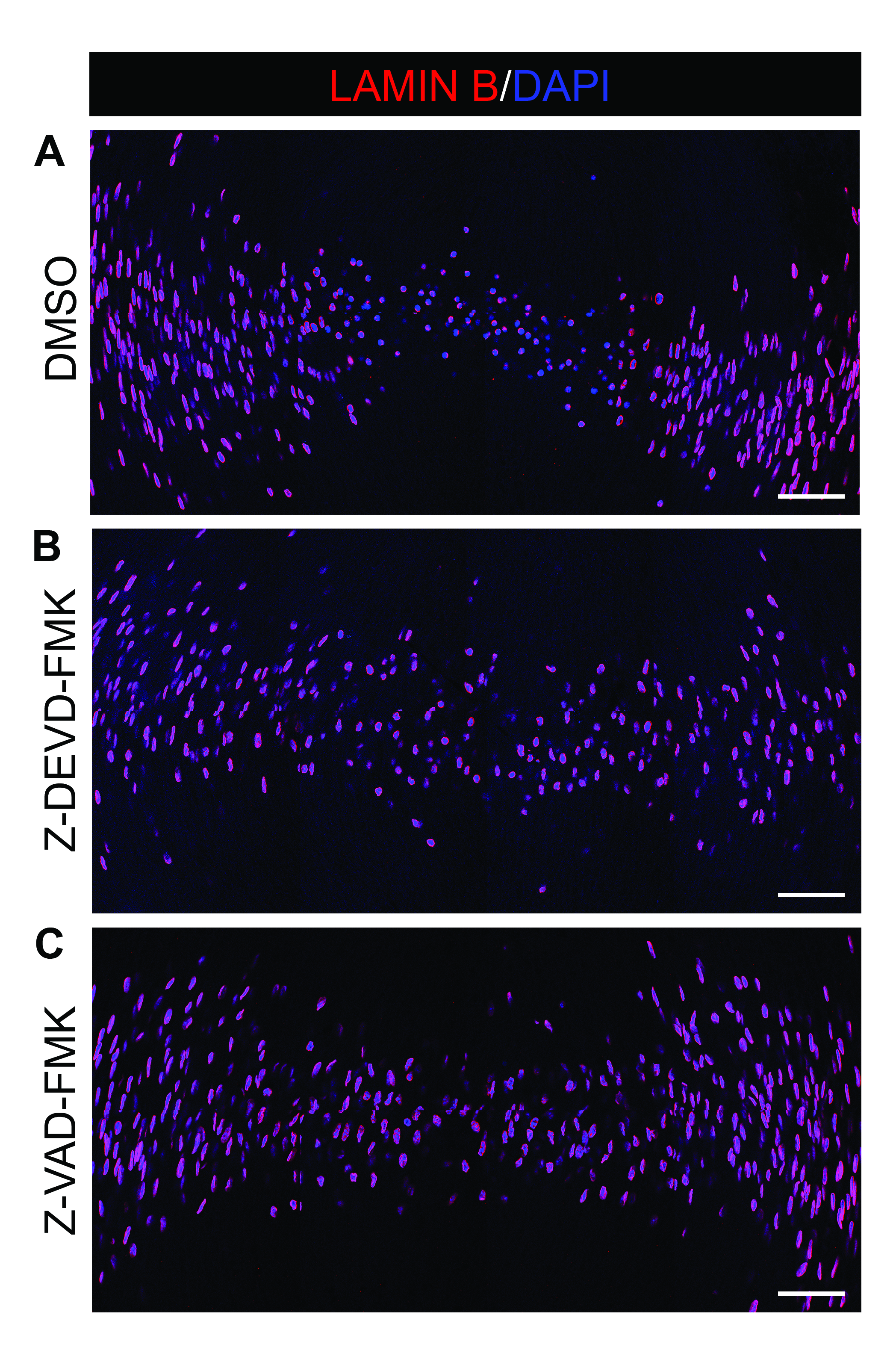

Supplement: Supplementary file 6 — Supplemental Figure 5 [file 41420_2023_1680_MOESM6_ESM.tif]

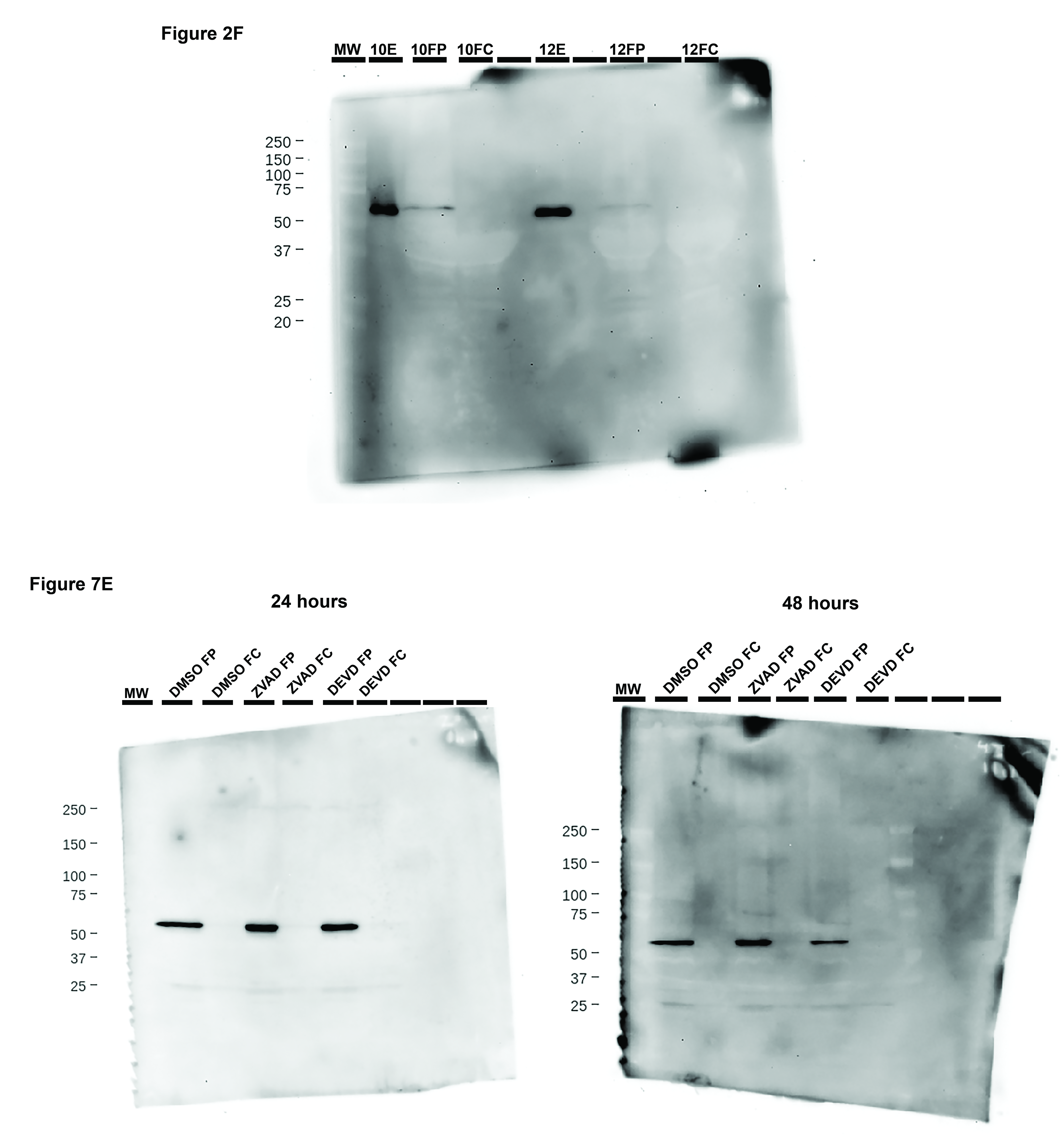

Supplement: Supplementary file 7 — Original Data File [file 41420_2023_1680_MOESM7_ESM.tif]
